# A case report of a novel *de novo* variant in *PPP2CA* causing a neurodevelopmental disorder and epilepsy

**DOI:** 10.3389/fpsyt.2026.1889298

**Published:** 2026-08-07

**Authors:** Lei Xu, Yanfeng Shen, Guixiang Zhang

**Affiliations:** Department of Rehabilitation Medicine, Dalian Women and Children’s Medical Center, Dalian, China

**Keywords:** ADHD, hypotonia, PP2A, PPP2CA gene, seizures

## Abstract

Neurodevelopmental disorders related to protein phosphatase 2 catalytic subunit alpha (*PPP2CA*) are currently recognized as Houge–Janssens syndrome 3 (OMIM: 618354), an autosomal dominant disorder characterized by developmental delay, intellectual disability, autism spectrum disorder, seizures, brain abnormalities, and hypotonia. Here, we report a patient with a *PPP2CA* variant. Trio-based whole-exome sequencing was performed on the patient and her parents, and a novel *de novo* heterozygous variant *PPP2CA* (NM_002715: c.218G > A, p.Gly73Asp) was identified, resulting in an amino acid substitution within the catalytic subunit. Clinically, the patient presented with epilepsy, hypotonia, attention-deficit/hyperactivity disorder, and intact intellectual functioning; there were no structural brain abnormalities. *PPP2CA* variants are rare, and the variant identified in this study has not been previously reported in the literature or public databases. Our findings support the pathogenicity of this variant and further expand the pathogenic variant spectrum of the *PPP2CA* gene. Furthermore, the observed genotype–phenotype correlation provides valuable information for prognosis and genetic counseling.

## 1 Introduction

Neurodevelopmental disorders (NDDs) are a heterogeneous group of rare disorders, including intellectual disability (ID), developmental delay (DD), and autism spectrum disorder (ASD). Disruption of gene expression during early brain development may impair normal neurodevelopment and contribute to the development of various NDDs. Recent large-scale exome- and genome-sequencing studies have substantially advanced our understanding of the genetic basis of NDDs. Protein phosphorylation is the most common post-translational modification, with more than 96% of phosphorylation events occurring on serine (Ser) and threonine (Thr) residues (1). Protein phosphatase 2A (*PP2A*) is the major serine/threonine phosphatase responsible for approximately 50% of Ser/Thr dephosphorylation events (2). *PP2A* plays critical roles in nervous system development and tumorigenesis by regulating multiple biological processes, including apoptosis, autophagy, cell proliferation, and DNA repair (3). The *PP2A* complex consists of a C subunit that possesses catalytic phosphatase activity, a regulatory B subunit that confers substrate specificity and regulation, and a structural A subunit that serves as a scaffold for C- and B-type subunits (4). To date, pathogenic variants have been identified in several *PP2A-*related genes, including *PPP2R5D*, which encodes the regulatory B56δ subunit; *PPP2R1A*, encoding the structural Aα subunit; and protein phosphatase 2 catalytic subunit alpha (*PPP2CA*), encoding the catalytic Cα subunit and harboring novel missense and loss-of-function variants (5, 6). *PP2A*-related neurodevelopmental disorders are characterized by DD, ID, ASD, abnormal head circumference, hypotonia, facial dysmorphism, and corpus callosum abnormalities, giving rise to clinically overlapping syndromes: Houge–Janssens syndrome (HJS) types 1, 2, 3, and 4 (7). Here, we report an additional patient with a *de novo PPP2CA* variant diagnosed with autosomal dominant HJS3. The identified variant results in a p.Gly73Asp amino acid substitution in the catalytic Cα subunit. Our findings support its pathogenicity and further expand the pathogenic variant spectrum of the *PPP2CA* gene. Furthermore, the observed genotype–phenotype correlation provides valuable information for prognosis and genetic counseling.

## 2 Materials and methods

### 2.1 Trio-based whole-exome sequencing

Trio-based whole-exome sequencing was performed on the patient and her parents. Peripheral blood samples (2 mL each) were collected from all three individuals, and genomic DNA was extracted using the Blood Genomic DNA Extraction Kit (Kangweishiji, Shanghai, China). Following DNA quality control and library preparation, target-region DNA fragments were enriched to construct the whole-exome library using the xGen Exome Research Panel (version 2.0) (IDT, Iowa, USA). High-throughput sequencing was performed on the MGISEQ-T7 platform (Huadazhizao, Beijing, China) at Beijing Chigene Translational Medicine Research Center Co., Ltd. (Beijing, China).

After quality control of the raw sequencing data, paired-end clean reads were aligned to the Ensembl reference genome (GRCh37/hg19) using the Burrows–Wheeler Aligner (BWA, version 0.59). Base quality score recalibration, as well as single-nucleotide polymorphism (SNP) and short insertion/deletion (indel) calling, was performed using the Genome Analysis Toolkit. Variants were filtered based on sequencing depth and variant quality to retain only high-confidence calls.

Variant annotation was performed using an online system independently developed by Chigene (www.chigene.org). The system annotates minor allele frequencies (MAFs) using multiple population databases, including the 1000 Genomes Project, dbSNP, ESP, ExAC, and the Chigene in-house MAF database. The pathogenicity of each variant was assessed according to the American College of Medical Genetics and Genomics/The Association for Molecular Pathology (ACMG/AMP) guidelines. For functional prediction of protein-coding variants, a suite of software tools was used, including PROVEAN, SIFT, PolyPhen-2, MutationTaster, M-Cap, and REVEL. The OMIM, Human Gene Mutation Database (HGMD), and ClinVar were used as reference for variant pathogenicity assessment. To predict the potential impact of variants on RNA splicing, MaxEntScan, dbscSNV, and GTAG were used instead of protein structure prediction tools.

## 3 Results

### 3.1 Clinical history

A girl aged 8 years and 9 months was admitted to our pediatric neurology department within 6 h of seizure onset. The seizure occurred during her sleep and was characterized by generalized tonic–clonic seizures. The seizure lasted approximately 20 min before resolving spontaneously. On admission, the patient was in good general condition, with no headache, fatigue, or fever, and her appetite and sleep patterns were normal. She was born at full term via vaginal delivery to a G1P1 mother. Her birth weight was 3,600 g, and her head circumference was 33 cm (- 0.4 SD) at birth and 45 cm (+ 0.7 SD) at 1 year of age. The mother experienced fetal cardiac rhythm abnormalities and intrauterine distress at 30 weeks of gestation. Both parents were healthy, with no family history of genetic disorders, epilepsy, or febrile seizures. The patient achieved head control at 3 months, independent sitting at approximately 9 months, and unassisted walking at 18 months, and was able to say “father” and “mother” at 1 year of age. Her early developmental milestones were within the normal range, but subsequent motor development lagged behind age-appropriate expectations. At present, she exhibits reduced motor endurance and impaired coordination, illegible handwriting, hyperactivity, poor concentration, impulsive verbal outbursts, and early academic difficulties. The patient also exhibits immature social behavior, has difficulty in maintaining peer relationships, and frequently experiences interpersonal conflicts.

### 3.2 Neuropsychological and behavioral assessments

At 2 years and 3 months of age, assessment using the Gesell developmental scale revealed the following developmental quotients (DQs): gross motor, 76; fine motor, 76; language, 103; adaptive behavior, 119; and personal–social, 87. These results indicated delayed gross and fine motor development. At 7 years and 9 months of age, assessment using the Wechsler Intelligence Scale for Children revealed a full-scale intelligence quotient of 83.1. Concurrently, the Swanson, Nolan, and Pelham-IV-Rating Scale revealed an inattention subscale score of 1.33 and a hyperactivity–impulsivity subscale score of 0.89. The Parent Symptom Questionnaire revealed an impulsivity–hyperactivity index of 1.75 and a hyperactivity index of 1.20.

### 3.3 Physical examination

The patient was conscious and alert, with appropriate responses to questions. However, she exhibited poor eye contact, excessive restlessness, and difficulty remaining seated. She had a cheerful facial expression, a head circumference of 51 cm (+ 1.1 SD), and a prominent upper lip. No abnormalities were detected on a gross examination of the cranial nerves. Muscle strength was normal in all limbs, but hypotonia was present in the lower limbs. Bilateral tendon reflexes were symmetrical, with knee hyperextension, heel valgus, and pes planus. The patient was right-handed, and the finger-to-nose test revealed impaired coordination.

### 3.4 Accessory examination

**Figure 1:**
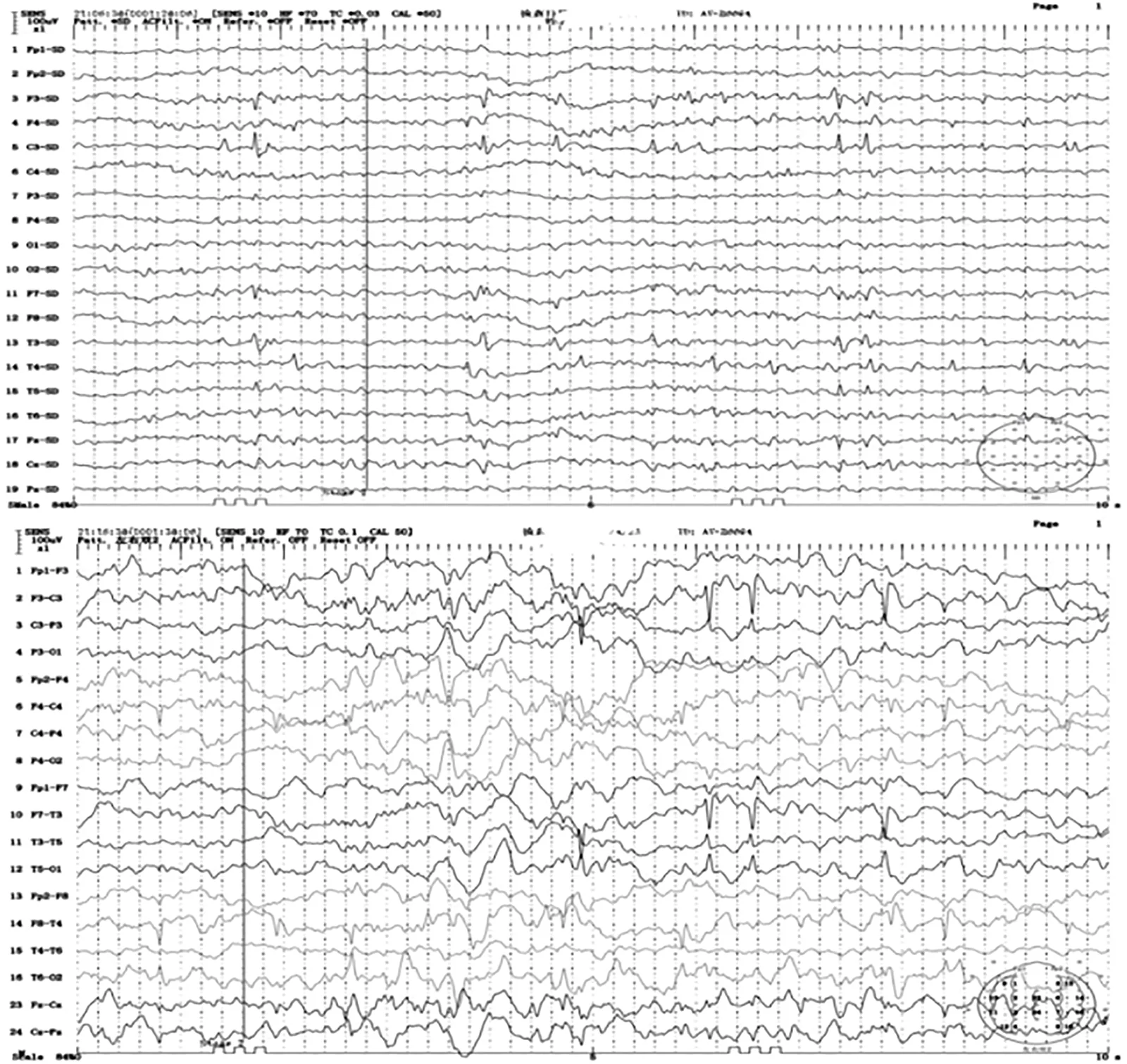
EEG findings: Scattered high-amplitude spike waves and 2.5–3.5 Hz spike-and-slow waves in the middle, posterior temporal, and central regions of both sides during wakefulness and sleep.

**Figure 2:**
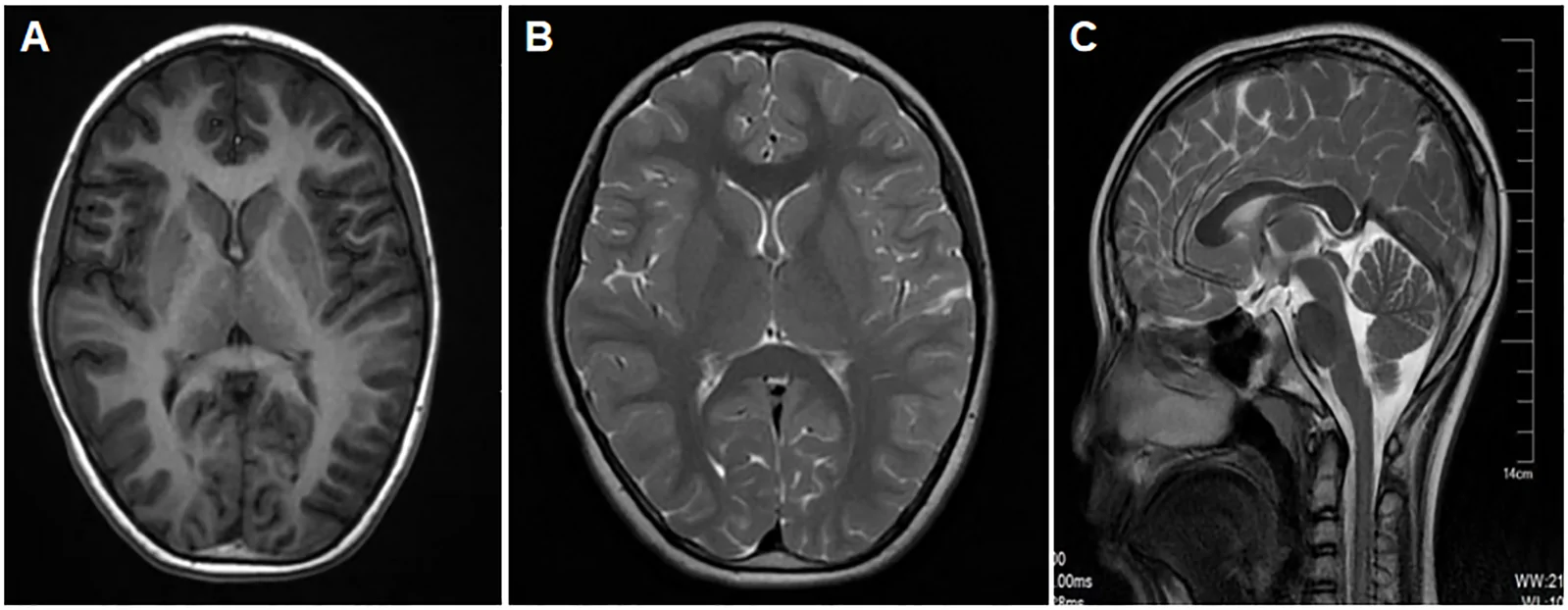
MRI brain scan revealing no significant abnormal signals, **(A)** T2 image of the axial section, **(B)** T2 flair image of the axial section, and **(C)** T1 image of the sagittal section.

The patient underwent extensive laboratory investigations, including a neuro-metabolic screen, homocysteine, blood ammonia, folic acid, vitamin B12, lactate, thyroid function tests, thyroid antibodies, complete blood count, coagulation profile, blood biochemistry, electrolytes, and blood glucose. All results were within normal limits. Blood amino acid and acylcarnitine analyses, as well as urine organic acid screening, were also within normal limits. The patient’s brain magnetic resonance imaging (MRI) revealed no significant abnormalities. Electroencephalogram (EEG) revealed asynchronous discharges during sleep and a discharge index of 30%–50% during non-rapid eye movement sleep. Scattered high-amplitude spike waves and 2.5–3.5 Hz spike-and-slow-wave complexes were observed in the bilateral mid-temporal, posterior temporal, and central regions during wakefulness (**Figures 1**, **2**).

### 3.5 Genetic findings from this study

**Figure 3:**
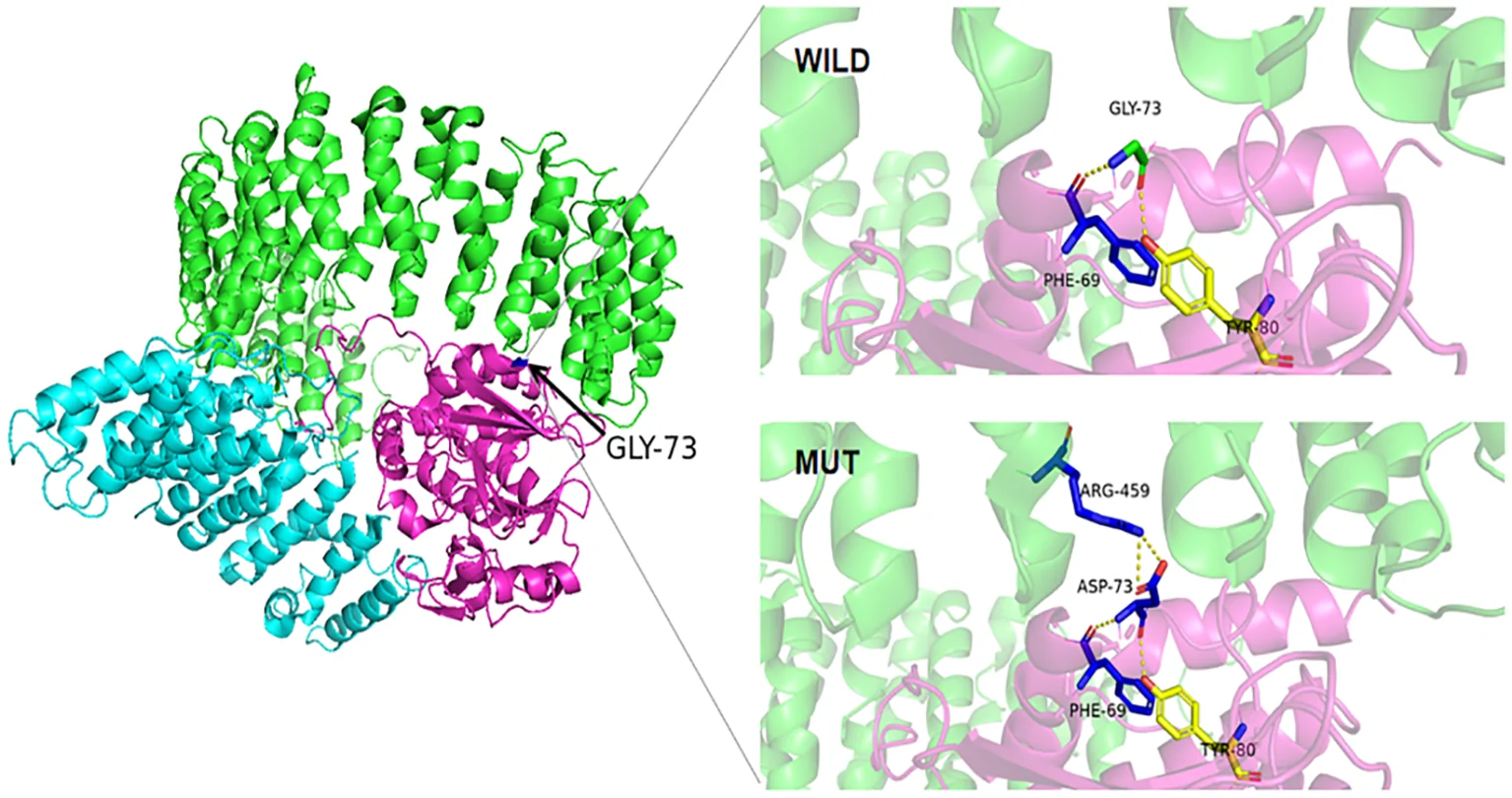
PyMOL analysis of the structural impact of the p.Gly73Asp mutation on the PP2A protein. In the crystal structure of protein phosphatase 2A (PP2A, PDB:2IAE), the subunits are visualized with the following color coding: green for the scaffold subunit A, blue for the regulatory subunit β, and purple for the catalytic subunit C. Dashed lines in the figure represent hydrogen bonds; WILD denotes the wild-type, and MUT denotes the mutant type.

We identified a novel missense variant in *PPP2CA* (NM_002715: c.218G > A, p.Gly73Asp) in the patient, resulting in a glycine-to-aspartic acid substitution at amino acid residue 73. Both parents were wild-type, indicating that the variant arose *de novo*. Notably, this variant was absent from major public databases (including ESP, the 1000 Genomes Project, gnomAD, and ExAC), as well from our in-house Chigene database. Multiple bioinformatics tools consistently predicted this variant to be functionally deleterious: PROVEAN (deleterious, - 6.52), SIFT (damaging, 0.0), PolyPhen-2 (HDIV/HVAR: probably damaging, 0.998/0.963), MutationTaster (disease-causing, 0.89), M-CAP (damaging, 0.187029), REVEL (deleterious, 0.521), CADD (Deleterious, 29.4), and AlphaMissense (pathogenic, 0.9946). According to the ACMG variant classification guidelines, this variant was classified as likely pathogenic (PS2, PM2-Supporting, PP2, and PP3). **Figure 3** illustrates the PyMOL analysis of the structural impact of the p.Gly73Asp variant on the PP2A protein (PDB: 2IAE). In the wild-type (WT) PP2A, glycine at position 73 (Gly73) in the catalytic C subunit contributes to the protein’s structural stability through a hydrogen-bonding network, forming hydrogen bonds with phenylalanine 69 (Phe69) and tyrosine 80 (Tyr80). Following the p.Gly73Asp substitution, Asp73 in the mutant (MUT) protein is predicted to form an additional hydrogen bond with arginine 459 (Arg459) of the scaffold A subunit. These alterations in the hydrogen-bonding network may lead to changes in interactions among *PP2A* subunits, thereby impairing the protein’s overall conformational stability. The *PPP2CA*-associated disorders are characterized by mild-to-profound ID, DD, seizures with or without brain abnormalities, hypotonia, ASD, and other major behavioral problems. These manifestations are consistent with the moderately affected clinical phenotype of the present patient.

### 3.6 Recent follow-up results

The patient was diagnosed with epilepsy. Additionally, she exhibited social interaction difficulties, hypotonia, learning difficulties, and attention-deficit/hyperactivity disorder (ADHD), with no evidence of brain abnormalities. Levetiracetam was initiated for seizure control, along with executive function training, behavioral therapy, and sensory integration therapy. Currently, the patient’s epilepsy is well controlled, and her academic performance is average. However, she continues to experience attention deficits, limited social skills, immature behavior, and frequent interpersonal conflicts.

## 4 Discussion

The *PPP2CA* gene is located on chromosome 5q31.1, spans approximately 32 kb, and contains seven exons. It encodes the catalytic subunit Cα of protein phosphatase 2A (*PP2A*), which contains phosphate-binding domains and interacts with the structural A and regulatory B subunits. These subunits contain HEAT repeats and form a heterotrimeric complex (8). As a major serine/threonine phosphatase, *PP2A* participates in multiple cellular signaling pathways and regulates processes such as cell cycle control, cellular differentiation, and tumor suppression (8). *PP2A* is a heterotrimeric enzyme composed of a catalytic C subunit, a structural A subunit, and a regulatory B subunit. The *PP2A* holoenzyme is ubiquitously expressed in various tissues, with particularly high expression in neurons, and is also present in astrocytes and oligodendrocytes (9). The *PP2A* catalytic C subunit is present in multiple subcellular compartments, including the cytosol, nucleus, microtubules, neurofilaments, membranes, and synaptosomes (9). The *PPP2CA* knockdown promoted the upregulation of phosphorylated mTOR expression (10). Brain-specific *PP2A* Cα knockout in mice resulted in microcephaly, cortical atrophy, and impaired learning and memory (11). Novel *PPP2CA* variants have been associated with a spectrum of clinical manifestations, including ID, DD, epilepsy, behavioral abnormalities, structural brain abnormalities, and hypotonia (14). Verbinnen et al. reported an 18-year-old female patient with a *de novo PPP2CA* missense variant (p.Cys196Arg). She presented with mild DD, relatively intact intellectual functioning, autistic features, normal muscle tone, no brain abnormalities, and mild dysmorphic features (12). To date, fewer than 20 *PPP2CA* variants have been documented in the HGMD, including missense, nonsense, frameshift, and synonymous variants. Biochemical characterization has demonstrated that most reported variants are truncating or loss-of-function variants, consistent with both haploinsufficiency and dominant-negative disease mechanisms (9). The patient harbored a heterozygous *de novo* Gly73Asp variant, which has not been previously reported. The clinical features of *PPP2CA*-related HJS3 include a broad spectrum of neurodevelopmental abnormalities, including mild-to-severe ID/DD, ASD in approximately 50% of patients, epilepsy in approximately 50% of patients, and structural brain abnormalities, such as microcephaly, macrocephaly, corpus callosum dysplasia, and progressive brain atrophy (15). Facial dysmorphism is typically mild or absent (12, 13). The patient reported here falls well within this clinical spectrum, presenting with epilepsy, hypotonia, behavioral abnormalities, ADHD, no brain abnormalities, and relatively intact intellectual functioning. The p.Gly73Asp variant identified in this patient is located in a highly conserved region. Structural modeling predicted that the variant site forms hydrogen bonds with neighboring amino acid residues, thereby contributing to the protein’s structural stability. The p.Gly73Asp substitution may disrupt these hydrogen-bonding interactions, altering the protein structure and impairing its function. This study evaluated the structural impact of the variant on *PP2A* using bioinformatic analyses and protein crystal structure modeling. However, no *in vitro* functional assays, phosphatase activity assays, or *in vivo* animal studies were performed to validate its pathogenic mechanism. Further functional studies are required to validate the effects of this variant on *PP2A* enzymatic activity and inter-subunit interactions.

## 5 Conclusion

This novel missense variant was classified as likely pathogenic according to the ACMG variant classification guidelines. Based on the patient’s clinical manifestations, including hypotonia, sensory integration dysfunction, behavioral abnormalities, and epilepsy, and combined with the genetic findings, the *PPP2CA* gene p.Gly73Asp variant was identified as the likely cause of the disorder. *PPP2CA*-related HJS3 is an extremely rare disorder, and the p.Gly73Asp variant identified in this study has not been previously reported in the literature or public databases. This novel variant expands the pathogenic variant spectrum of the *PPP2CA* gene. A review of available national and international literature indicates that nearly all previously reported pathogenic *PPP2CA* variants have arisen *de novo*, consistent with the inheritance pattern observed in our patient. Notably, the clinical phenotype of our patient differs in part from those previously reported, further expanding the recognized phenotypic spectrum of *PPP2CA*-related neurodevelopmental disorders.

## Data Availability

The original contributions presented in the study are included in the article/supplementary material, further inquiries can be directed to the corresponding author/s.
